# Cost of dialysis therapies in rural and remote Australia – a micro-costing analysis

**DOI:** 10.1186/s12882-019-1421-z

**Published:** 2019-06-25

**Authors:** G. Gorham, K. Howard, Y. Zhao, A. M. S. Ahmed, P. D. Lawton, C. Sajiv, S. W. Majoni, P. Wood, T. Conlon, S. Signal, S. L. Robinson, S. Brown, A. Cass

**Affiliations:** 1 Renal Program, Wellbeing & Preventable Chronic Diseases Division, Menzies School of Health Research, Darwin, Northern Territory Australia; 2 Sydney School of Public Health, University of Sydney, Sydney, New South Wales Australia; 3 Department of Health, Northern Territory Government, Darwin, Northern Territory Australia; 4 Western Desert Nganampa Walytja Palyantjaku Tjutaku Northern Territory, Alice Springs, Australia; 5 Flinders University Northern Territory Medical Program, Darwin, Northern Territory Australia; 6 Primary Health, Hobart, Tasmania Australia

**Keywords:** Dialysis, Aboriginal, Rural, Remote, Costs, Expenditure

## Abstract

**Background:**

Maintenance dialysis is a costly and resource intense activity. In Australia, inadequate health infrastructure and poor access to technically skilled staff can limit service provision in remote areas where many Aboriginal dialysis patients live. With most studies based on urban service provision, there is little evidence to guide service development. However permanent relocation to an urban area for treatment can have significant social and financial impacts that are poorly quantified. This study is part of a broader project to quantify the costs and benefits of dialysis service models in urban and remote locations in Australia’s Northern Territory (NT).

**Methods:**

We undertook a micro-costing analysis of dialysis service delivery costs in urban, rural and remote areas in the NT from the payer perspective. Recurrent maintenance costs (salaries, consumables, facility management and transportation) as well as capital costs were included. Missing and centralised costs were standardised; results were inflated to 2017 values and reported in Australian dollars.

**Results:**

There was little difference between the average annual cost for urban and rural services with respective median costs of $85,919 versus $84,629. However remote service costs were higher ($120,172 - $124,492), driven by higher staff costs. The inclusion of capital costs did not add substantially to annual costs. Annual home haemodialysis costs ($42,927) were similar to other jurisdictions despite the significant differences in program delivery and payment of expenses not traditionally borne by governments. Annual peritoneal dialysis costs ($58,489) were both higher than home and in-centre haemodialysis by recent national dialysis cost studies.

**Conclusion:**

The cost drivers for staffed services were staffing models and patient attendance rates. Staff salaries and transport costs were significantly higher in remote models of care. Opportunities to reduce expenditure exist by encouraging community supported services and employing local staff. Despite the delivery challenges of home haemodialysis including high patient attrition, the program still provides a cost benefit compared to urban staffed services. The next component of this study will examine patient health service utilisation and costs by model of care to provide a more comprehensive analysis of the overall cost of providing services in each location.

**Electronic supplementary material:**

The online version of this article (10.1186/s12882-019-1421-z) contains supplementary material, which is available to authorized users.

## Background

Kidney disease is a significant health issue globally and disproportionately impacts disadvantaged populations [1–3]. In Australia there is a steep gradient in prevalence from urban to remote areas with people in remote areas suffering a much heavier burden of kidney disease [4]. Nationally, Aboriginal Australians are more likely to be affected by kidney disease. In the Northern Territory (NT), where the majority (70%) of Aboriginal people live in remote areas, incidence and prevalence rates are the highest in Australia [5]. This picture of high kidney disease incidence and prevalence rates amongst First Nation people in remote areas, is mirrored in other developed countries such as Canada, New Zealand, USA and parts of South Africa [6, 7]. These countries have similarly vast remote regions, difficult to reach service locations, shared colonial histories and in the case of Canada and New Zealand, comparable health care systems with Australia [8, 9].

Australia has a publicly funded health care system predominantly based on the delivery and re-imbursement of defined activities [10]. The Australian Government reimburses State and Territory hospitals for the type and mix of services delivered and the provision of dialysis is the responsibility of each State or Territory. However, concerned by potential costs and sustainability of low volume remote area services, most jurisdictions centralised dialysis services.

The NT has a relatively small population (less than 230,000 people) sparsely dispersed over a large geographical are (1.3million square kilometres) [11]. Dialysis patients come from more than 70 remote and very remote communities including those across jurisdictional borders. Remote service delivery is challenged by the high number of required service locations and multiple jurisdictional responsibilities. Costs are also amplified by limited remote infrastructure, expensive transport options and difficulties in recruiting and retaining skilled staff. Consequently, there are few staffed dialysis services in remote areas and most people must relocate to access treatment. However, the full costs of providing staffed services in remote locations are largely unknown with few studies available to provide guidance to policy makers on costs [12, 13]. Most cost studies focus on dialysis provision in urban areas in developed countries or where significant economies of scale have been achieved in developing countries [14–16], or on comparative costs of home dialysis vs other modalities [17, 18].

The lack of staffed dialysis services in remote communities limits the opportunities for a patient to have treatment at home to self-care haemodialysis (HD), peritoneal dialysis (PD) and transplantation. However, transplantation rates amongst Aboriginal people are low, and PD and home HD make up less than 10% of all people requiring treatment. The demand for staffed services in the NT is therefore high at 75% of all kidney treatments. This is consistent with studies of First Nation people in Canada and New Zealand [5, 12], but is in stark contrast to other Australian jurisdictions, where care in a staffed facility comprises 40% of all treatments [5].

Dialysis is a long-term treatment that can extend into decades. In the NT, as most staffed services are centralised in urban areas, permanent relocation for medical treatment and the subsequent dislocation, can have significant consequences for the individual as well as for government services. This is particularly stressful for Aboriginal people who are more likely to be affected at a younger age and when they are at their most active in their working and family life [19]. The provision of an accessible and culturally acceptable service is therefore paramount to patients [20, 21].

In response to escalating demand for dialysis treatments from the early 1990s, the NT government implemented measures aimed at quickly increasing capacity while containing costs. This perpetuated a model of large, centralised, urban services [22]. While successive governments supported services closer to home, the model was primarily one of self-care, thus nearly 85% of Aboriginal dialysis patients still needed to relocate permanently to access treatment [23]. The establishment of staffed remote services therefore emerged from independent action by patients and their remote communities to address the requirements for relocation [24]. Over the intervening 20 years, this approach resulted in a rather unique dialysis model of care that was community determined and driven. It also resulted in the development of partnerships between community owned organisations (eg Purple House, see Additional file 2.) and governments to support staffed remote services [22].

This study forms part of a broader mixed methods project using linked administrative data to examine the costs and benefits of various models of dialysis care in the NT and more widely in Australia [25]. The findings reported here address the knowledge gap related to dialysis service delivery costs in urban, rural and remote areas through a detailed analysis of expenditure of staffed facilities and self-care HD and PD in the NT.

## Methods

### Study setting

This analysis focusses on dialysis service delivery costs from the payer perspective (DoH and PH). At the time of this study the partnership between the NT Department of Health (DoH) and Purple House (PH) consisted of the DoH providing PH a fixed annual amount and in-kind contribution for each dialysis treatment, equivalent to medical supervision, consumables and maintenance support available to a self-care patient. This was in line with the DoH policy of supporting services closer to home through self-care. PH covered all other expenditure for service delivery (staff, infrastructure, transport, essential services) [26]. The cost of maintenance (regular) dialysis delivered in hospitals were not included in this study as these services are co-located with acute services. The separation of costs was considered beyond the scope of this analysis.

At the time of the analysis, the NT was caring for more than 725 patients with end stage kidney disease. The mean age of patients was 45 years with a slightly higher proportion of females to males. A small proportion of patients were undertaking self-care HD and PD (less than 10%) and approximately 13% were living with a transplant [27]. Most patients received more than one treatment modality (transplantation, HD or PD) in the 12-month period. By far, the majority of patients (530) received care in a staffed facility and for many, at more than one location (urban, rural and remote) in the 12-months. All facilities except the remote staffed services were at capacity.

Renal services in the NT are organised on a notional “hub and spoke” model, where the “hub” or tertiary provider oversees service delivery by other dialysis services. In the NT, larger urban staffed facilities function as hub services. We examined expenditure by location and service type, allocating the service to one of five dialysis models of care (DxMoC) described in detail in Table 1.

**Table 1:** Dialysis models of care in the Northern Territory

Dialysis Model	Description	Characteristics	Access Limitations
DxMoC1 Urban staffed service (Hub and non-hub)	Both services provide maintenance haemodialysis; Hub service also supports pre-dialysis care and training and support for self-care therapies.	Larger facilities in urban areas; default service when others at capacity; for complex patient issues.	*No criteria*: all patients commence treatment here while waiting training or space in more remote unit.
DxMoC2 Rural staffed service	Maintenance haemodialysis; some support for self-care patients. 350-500 km from hub by road.	Smaller more distant facilities; often co-located with local hospitals to access support services.	*Criteria*: stable patients adhering to treatment; usually a waiting list.
DxMoC3 Remote staffed service (Government)	Maintenance haemodialysis; some support for self-care patients. 80 km by air from hub	Small units very distant from hub.	*Criteria*: patients clinically well, physically mobile, adherent with treatment. Issues with local recruitment. FIFO staff model
DxMoC4 Remote staffed services (Purple House)	Maintenance dialysis in home community with social supports. 80 – 1000 km from hub service	Aboriginal owned and community driven services; small units in remote areas; permanent and respite services to select (local) patients.	*Criteria*: Patient acceptance criteria less restrictive as more support services available. Rotating staff model
DxMoC5 Self-care dialysis	Training and support for self-care dialysis. Modalities include haemodialysis and peritoneal dialysis.	Very small dedicated multi-user facilities established for self-care haemodialysis in remote areas. Peritoneal dialysis attended at home.	*Criteria*: clinically stable, deemed competent and safe to deliver own care. Maintenance of equipment and facilities and assistance with in-community deliveries can be a challenge.

*DxMoC Dialysis Model of Care, FIFO Fly-in Fly-out*

### Terminology

This study uses geographic descriptors for dialysis locations consistent with local views and vernacular e.g. “urban”, “rural” and “remote”. Australia uses a nation-wide framework to systematically classify the relationship between geography, population and services access [28]. However, the framework terminology does not always equate to local usage. Thus, there are some discrepancies between our study location designations and the official Australian classifications for remoteness which classifies the majority of NT locations as remote or very remote (Additional file 1).

### Study design

#### Data sources

Financial data from the DoH included expenditure reports for all renal related cost centres and Service Level Agreements with PH for 2013/14. Direct and overhead expenditure incurred by the DoH on behalf of PH (e.g. dialysis treatments, medical/pharmacy consumables and specialist medical staff) were calculated and added to the cost model. Pharmacy costs were based on the cost calculation for DxMoC1, as patients rotated through both services. PH also provided Profit and Loss statements for 2016/17 allowing independent analysis of the income and expenditure attributed to dialysis services (personnel, most variable eg transport, essential services and fixed costs). All costs have been reported in 2017 Australian dollars.

Capital costs for each service were based on original construction costs or recent construction costs of facilities of similar size and location. Equivalent annual costs were calculated for capital costs based on the working life of the capital (provided by DoH) of 40 years or less, assuming no resale value and a 5% discount rate [29].

#### Dialysis cost components

We used a micro-costing approach and analysed service costs for *Direct* and *Shared* resources*.* Within the *Direct* cost of service provision, expenditure was separated into Salary, Variable Costs and Fixed Costs. Allocation of *Shared* costs e.g. medical, allied health and educator staff working across a number of sites or models of care, were calculated for each model according to time allocations as advised by the relevant staff. Other overhead costs (e.g. corporate services) were based on nationally derived values [30]. One-off costs for surgical creation of dialysis access were not included.

#### Assumptions and missing data

As the data were from different time periods and sources, to enhance comparability across models of care, we assumed consistent values for some items based on available and representative data provided by DoH. This included year 2017 processing costs (linen, waste removal), service floor plans and facility life spans based on construction materials and locations.

The Australian Government subsidises the cost of most medications for all residents through the Pharmaceutical Benefits Scheme [31]. Those with concession cards (pensioners, sickness benefits, unemployed), these medications are further subsidised leaving a small co-payment. Recognising the low socio-economic status of many renal patients, the DoH covers the cost of this co-payment. Therefore, there are no out-of-pocket costs for treatment and medications for renal patients in the NT.

Medication costs were estimated from 12 months of dispensed scripts; unit costs were from the Pharmaceutical Benefits Scheme Dispensed Price for Maximum Quantity (DPMQ) costs [31]. Erythropoietin stimulating agents were costed separately using DPMQ costs with usage based on expert clinical advice on proportions of patients receiving each agent, and dosing frequency.

Pathology costs were based on the total type and number of blood samples taken in a year and calculated on Medicare Benefits Schedule (MBS) fees for service items [32].

The availability of staff accommodation in rural and remote sites is critical to service sustainability. Accommodation is often subsidised (rural) or provided (remote) but entitlements were not offered consistently in our study by service providers, making comparisons difficult. The cost of providing staff accommodation were not included in the cost study.

Costs were adjusted to a base year of 2017 Australian dollars using the Australian Institute of Health and Welfare (AIHW) Health Price Index [33] unless otherwise specified. The line items, component inclusions and adjustments, are outlined in Table 2. Some costs were based on the National Efficient Price (NEP) derived from the activity-based funding re-imbursements [30].

**Table 2:** Line Items, sources and adjustments

Line Items	Components	Source and adjustment of $ values
*Salary*		
Nursing PCA /Admin staff	Overtime, penalty rates, higher duties, leave oncosts (superannuation), training, conference, workers compensations, recruitment, security clearances, relocation expenses and Agency/labour hire fees	Ledger – increase by 9% based on published annual salary increases [47]
*Variable costs*		
Operational Stores	Stationary, paper towels, cleaning products etc.	Ledger - HPI
Treatments	Treatment cost for dialysis (machine, R&M, water testing and consumables)	2017 cost/tr provided by hospital
Food Services	Patient meals, snacks plus high protein drinks	2017 cost/tr provided by hospital
Linen	Sheets, towels, patient gowns	2017 cost/kg/tr
Medical Stores	Medical consumables such as dialysis packs, gases, syringes, gauze, tapes etc	2017 cost/tr provided by hospital
Pharmacy	All dialysis drugs (saline, iron, heparin, lignocaine etc) EPO and outpatient medications	2017 PBS DPMQ costs
*Fixed costs*		
Information Technology	Phones, computers, internet, paging system, email	Ledger - HPI
Patient transport	Combination of taxi vouchers, rental/lease of mini bus and driver salaries	Ledger - HPI
Staff Transport	Leased vehicles, car hire, taxi vouchers and fuel and flights	Ledger - HPI
Cleaning	Cleaning services	Ledger – average cost/square metre - HPI
Freight	Intra-Territory and interstate transport costs	Ledger - HPI
Waste removal	Contract costs	Ledger – average cost/kg/tr HPI
Essential Services	Power, water and sewage	2017 cost/pt./tr provided by hospital
Facility R&M	Property/ground maintenance, pest eradication, Fire and Security services, etc.	Ledger - HPI
Depreciation/Leasing	Plant eg generator and facility leasing	Ledger - HPI (Leasing substituted for capital)
*Medical*	Nephrologists, Registrars, visiting specialists – salaries, sessional fees, travel	Ledger - Published annual salary increases [47]
*Allied Health and Training*	Social worker, Dietitian, Aboriginal Liaison Officers, nurse educators – salaries and travel	Ledger - Published annual salary increases [47]
*Pathology*	Total scheduled number and type of pathology tests in one year	2017 - MBS service fee per item
*Imaging*	Radiology	National Efficient Price 2013–14 - HPI
*Oncosts*	Corporate support	National Efficient Price 2013–14 - HPI
*Capital depreciation*	Construction costs for urban, rural, remote and relocatable facilities	Annuity factor [29] applied Life span - 40 yrs. Urban, 30 yrs. rural and remote and 25 yrs. relocatable facilities

*DPMQ Dispensed Price Maximum Quantity, EPO erythropoietin, HPI Health Price Index, kg kilogram, MBS Medicare Benefits Schedule, PBS Pharmaceutical Benefit Scheme, PCA Patient Care Assistant, pt Patient, R&M Repairs and maintenance, tr Treatment*

#### Calculation of costs

The aim of the micro-costing approach was to identify the average cost per treatment (CPT) for each model of care identified in Table 1. This was calculated by dividing the total expenditure for a model of care by the number of treatments completed over the same time period for HD or in the case of PD, by the number of patients. Treatment numbers for each year by service and model of care were provided by the DoH. Multiplying the CPT for each model of care by 156 estimated the annual cost per patient and assumed few patients were *prescribed* dialysis treatments more or less than three times per week. PD costs were calculated per patient per year.

## Results

Overall, and after adjustments, we found that the annual costs of service delivery in urban and rural areas were similar but remote based services were significantly higher. The co-location of facilities with other health services conferred some cost benefits through shared infrastructure and systems including storage, supply lines and facility management. The rural and remote models were more likely to sustain higher nursing costs related to staff entitlements for remoteness and recruitment, relocation and agency fees. Costs for visiting staff from the hub services had relatively small impacts.

Variation within models related to different staffing ratios, waste management practices (local or interstate services) and staff and patient transport costs. Salaries made up more than 50% of total costs and were also the main driver for cost differences between staffed models followed by capital depreciation. All models were particularly sensitive to low volume services.

### Profile of Urban Services (DxMoC1)

Four urban services (DxMoC1), two based in the Top End and two in Central Australia, treated the majority of NT dialysis patients. Only one DxMoC1 in each region acted as a hub service. In the Top End, the hub service oversaw the management of a smaller urban facility that sat within a larger health complex 30 km away. Staff were rostered from the hub service and often allocated on an ad hoc basis to the smaller facility. Consumables also flowed through the hub service.

In Central Australia, the hub service was co-located with other health services in a single health complex with some expenditure allocated on a cost sharing basis. This model included costs not evident at other services such as security and vehicles for patient transport. A second DxMoC1 facility was contracted as a public/private partnership. This facility delivered dialysis services for the NT government on a ‘price per treatment’ (PPT) basis where the PPT covered infrastructure, salaries and operational costs of the service. Payment was based on completed treatments irrespective of total patient numbers or staffing levels at the facility. Some services provided by the DoH (e.g. pharmacy items) but reimbursed by the contractor could not be captured as funds were absorbed into general revenue. Therefore, it is likely costs for this service were slightly overestimated. Medical staff, Allied Health, and non-dialysis pharmacy costs not included in the PPT were calculated as per other models. Depreciation was not calculated for this model, and line item comparisons could not be undertaken as these costs were included in the PPT. These costs have been represented as variable costs as they fluctuated according to the number of treatments completed.

Infrastructure costs for DxMoC1 were based on recent construction costs and calculated according to the size of the facility. Depreciation was annuitised over 40 years using a discount rate of 5%.

**Fig. 1:**
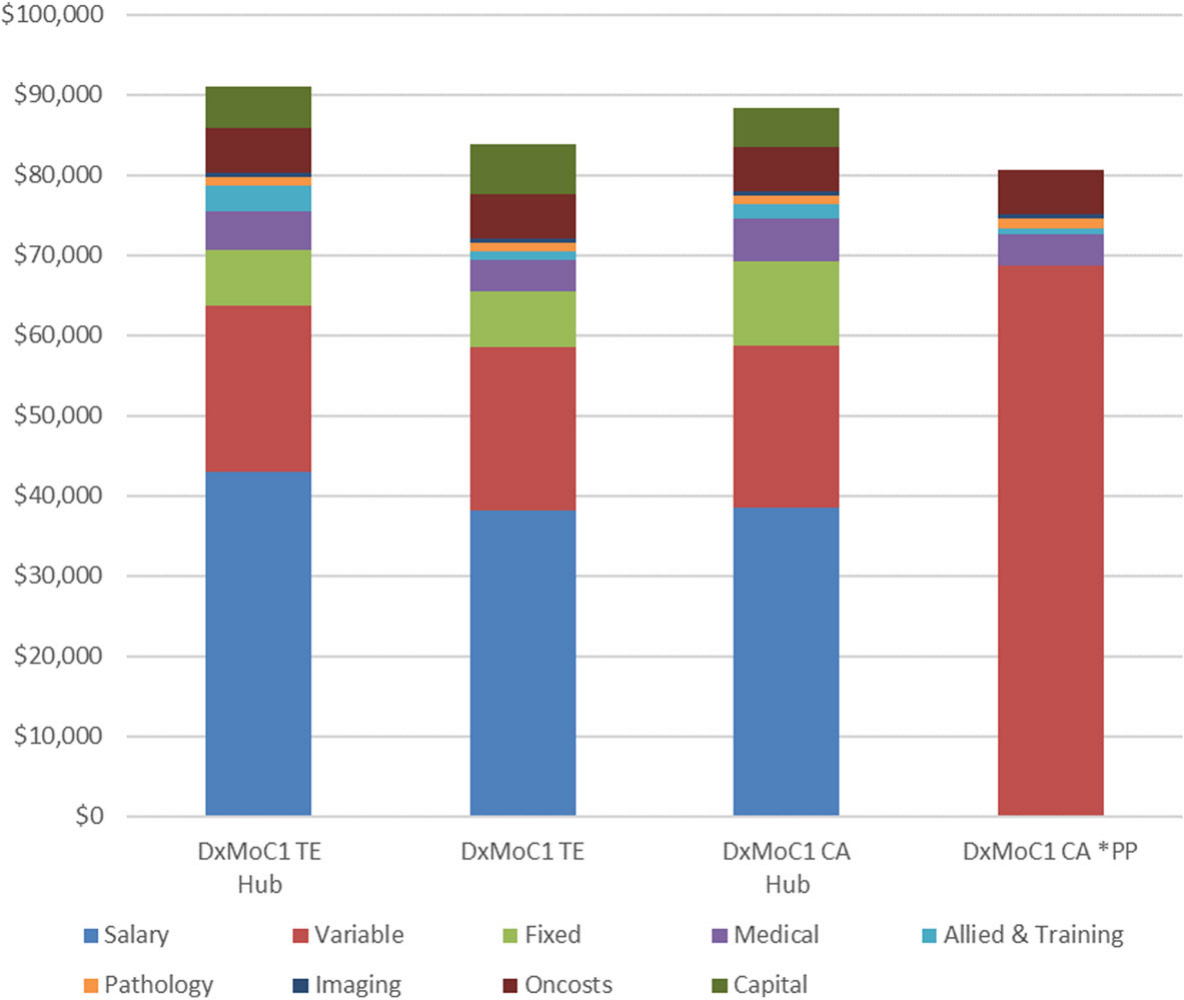
Annual average per patient costs by DxMoC1 Urban Services*. CA: Central Australia, PP: Public/private partnership – contracted service; TE: Top End*

Average annual cost per patient varied from $80,566 to $91,101 for DxMoC1 with a median per patient per year cost of $85,919. The Top End hub service had the highest annual per patient cost at $91,918 while the Central Australia contracted service (public/private partnership) had the lowest annual cost at $80,566 (Fig. 1).

### Profile of rural services (DxMoC2)

**Fig. 2:**
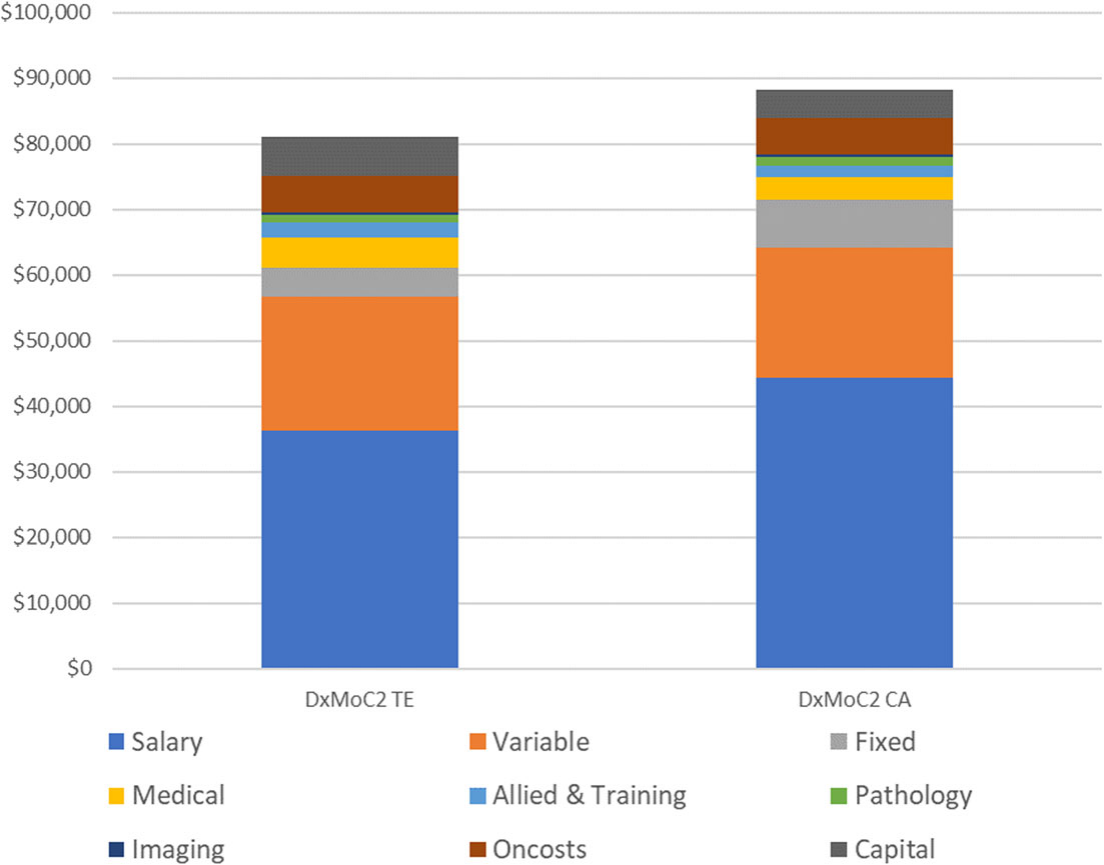
Annual average per patient costs - DxMoC2*. CA: Central Australia; TE: Top End*

Providing services ‘closer to home’, although a key strategy for the DoH, had been difficult to implement due to the very remote locations where services were required. Infrastructure costs and sustainability – primarily related to the ability to attract and retain skilled staff, were limiting factors. Consequently, the two rural services (DxMoC2) were co-located with local hospitals in an attempt to ameliorate these factors. At the time, they were one third the size of their urban counterparts. These smaller services tended to operate with lower ancillary staff, such as patient care assistants, administration staff and nurse managers. While staff costs were the main cost driver, the Central Australia DxMoC2 experienced higher costs related to recruitment, relocation and remote area allowances equating to a 13% difference in the overall salary costs. Annuitisation of capital costs, based on recent facility construction costs, assumed a 30 years working life and a 5% discount rate. The average annual cost per patient was $81,009 for the Top End and $88,249 for Central Australia with a median annual cost of $84,629 for DxMoC2 (Fig. 2).

### Profile of remote services (DxMoC3 and DxMoC4)

#### Government managed – DxMoC3

The delivery of remote staffed services (DxMoC3) in the NT commenced in 1998 with the establishment of a six-station facility on an island 80 miles off the coast of the NT. The stand-alone facility is independent of other health services on the island and remains the only remote service fully managed by the government in the NT. Care was overseen by the Top End hub service (DxMoC1) and renal nursing and ancillary staff rotated via air from the mainland on a weekly or more frequent basis. A weekly barge service provided transport to and from the island for equipment, consumables and waste. Some administrative activities (recruitment, coordination of rosters, flights, deliveries), borne by the DxMoC1 hub service, were difficult to track and therefore it is likely that the full costs of service delivery for this model were under-estimated.

#### Purple house - aboriginal community-controlled services – DxMoC4

At the time of the study, the PH provided dialysis services in several remote areas (DxMoC4) in partnership with the DoH. As noted, services were not contracted by the DoH but they provided funding support in a small annual grant and ‘in-kind’ contribution for each treatment delivered, equivalent to the consumables provided to self-care patients. PH is overseen by a Board of lay Directors made up of Traditional Land Owners and Elders. The service is staffed by a multidisciplinary team of paid professionals and volunteers. All dialysis is provided by trained nursing staff and the organisation is accredited with the required Australian Health Care bodies.

Historically the model was one of respite. Select relocated patients attending the urban government service (DxMoC1), were offered short periods of staffed dialysis care at home (DxMoC4). However, the success of PH in sourcing alternative government and philanthropic funds, as well as greater support from Traditional Land Owners, saw expansion of DxMoC4 sites across the NT. PH also operates on a hub and spoke model with the urban base in Central Australia managing remote service delivery.

While both DxMoC3 and DxMoC4 were delivered in remote and very remote areas, DxMoC3 was a single stand-alone facility, whereas DxMoC4 delivered services in smaller facilities over many sites, including the border communities of Western Australia. Costs were based on the aggregated data of eight sites for DxMoC4.

#### Cost drivers

Driven predominantly by staff costs, annual costs per patient for both services were high compared to DxMoC1 and DxMoC2. Staff in remote areas attract higher salaries and allowances compared to similar positions in urban areas due to the requirement to operate independently. Salaries made up 57% of overall costs but staff travel costs were significantly higher at DxMoC3, driven by the staffing model which involved weekly or more frequent rotations from the urban service. In contrast DxMoC4 staff stayed in the community for weeks to months at a time.

However, variable costs for DxMoC4 were more due to the consumable cost differences for the dialysis treatments. As DxMoC4 delivered the majority of their services from low volume two station facilities, treatment costs, which included dialysis machinery and maintenance, were treated the same as home HD, which was charged at a higher rate.

Annuitisation of capital costs assumed a life span of 30 years for DxMoC3, a brick and mortar facility, while DxMoC4 capital costs were calculated on eight relocatable facilities with a life span of 25 years. Despite the different infrastructures, the annual equivalent costs for the DxMoC4 aggregated sites were similar to DxMoC3, which was sensitive to treatment volume.

**Fig. 3:**
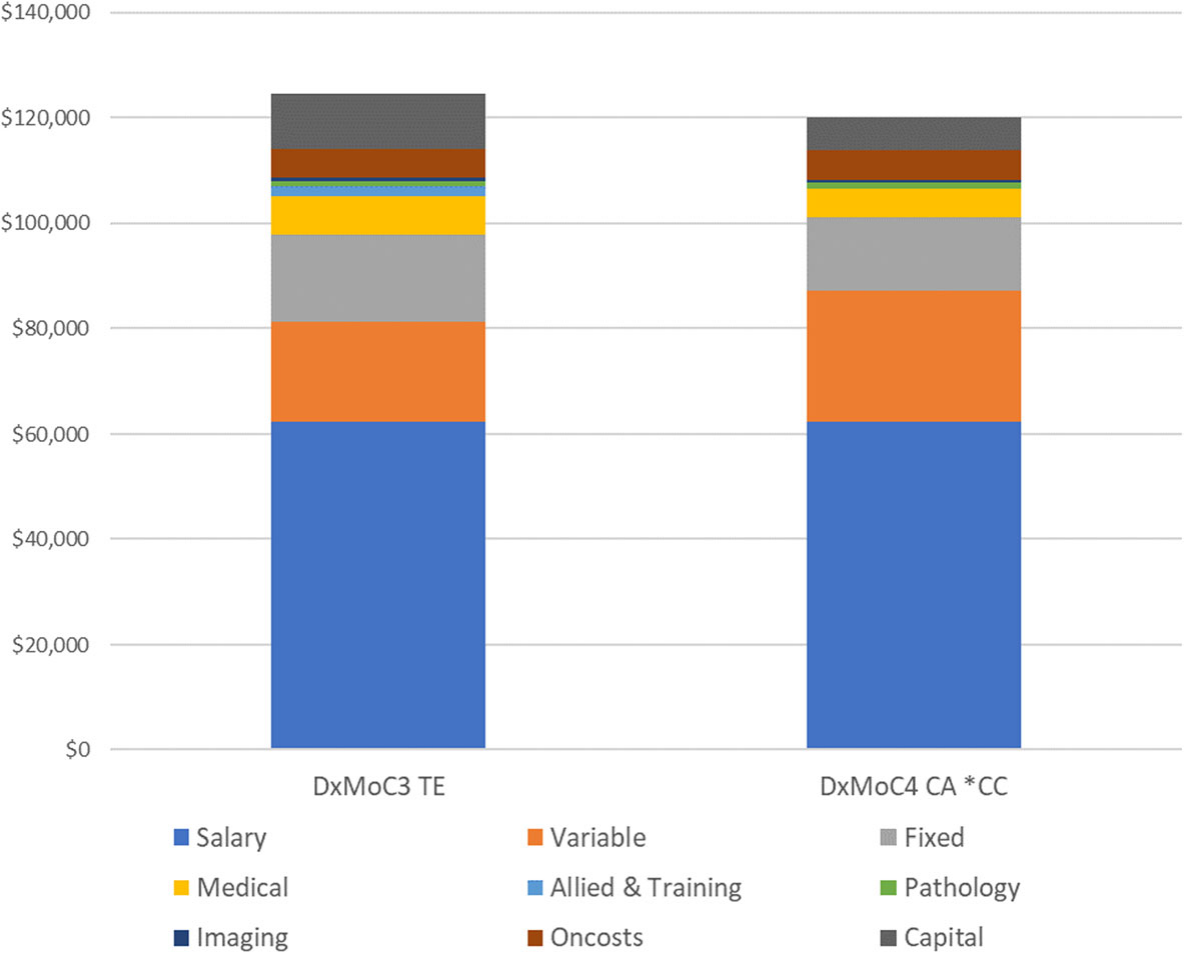
Annual average per patient costs – DxMoC3 and DxMoC4 Remote Sites*. CA: Central Australia, CC = community controlled; TE: Top End*

Overall (Fig. 3), there was little difference between the annual per patient costs of DxMoC3 ($124,679) and DxMoC4 ($120,215).

### Profile of self-care therapies (DxMoC5)

Self-care therapies are generally considered the preferred treatment option from a financial and quality of life perspective, although they are also noted to be potentially more burdensome for some patients and carers [34–38]. In the NT, the uptake has historically been low, below 10%, with significant variation between the Top End and Central Australia. Due to the low numbers of patients on self-care therapies in Central Australia, and the inability to make reliable comparisons, only the results from the Top End have been reported [5].

There were significant differences in the delivery of the home therapies program in the NT compared to other jurisdictions. Most patients were Aboriginal and returned to the remote area once trained. Training periods tended to be longer due to language differences, lower literacy and numeracy levels, unfamiliarity with technology and inconsistent attendance. Frequent home visits to patients were a key feature and therefore staff ratios were higher. Self-care HD also included infrastructure costs. The specifically designed ‘two station’ treatment facilities were provided by the government in recognition of the poor state of many homes in remote communities.

#### Home Haemodialysis

The average self-care HD training time was 6.1 months (2 to 12 months) for Aboriginal people and 4.8 months (3 to 7 months) for non-Aboriginal people, with approximately 30% of both groups withdrawing from the program after 2.8 months. Staff to patient ratios for self-care HD training were 1.43 staff for four patients in training. Staff travel costs were comparatively high for both the training component (exploratory and consultative remote site visits), and when patients were established at home (to check conditions and techniques). Fixed costs associated with the management of the dedicated HD infrastructure included leasing fees, rates and cleaning; costs generally not incurred in other jurisdictions.

**Fig. 4:**
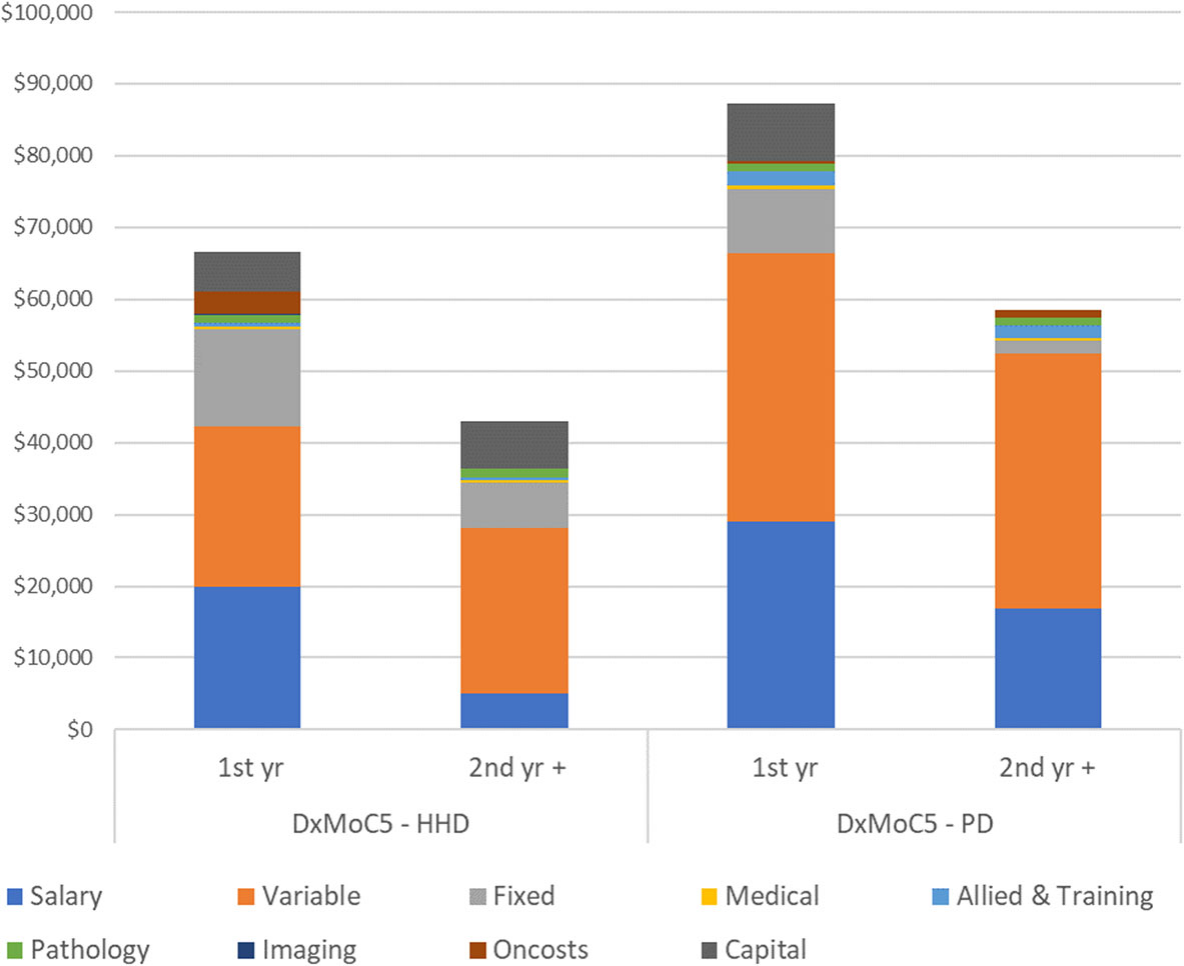
Annual per patient costs for 1st and 2nd year DxMoC5 – HHD and PD*.* HHD: home haemodialysis, PD: Peritoneal dialysis

Equivalent annual costs were estimated for capital costs for both the training facility and remote sites (assuming a 25 years working life) and calculated on a cost per treatment basis. Remote sites assumed four patients in attendance. Average annual costs per patient for home HD were $66,639 in the first year and $42,927 in the second and subsequent years (Fig. 4).

#### Peritoneal Dialysis

PD training was delivered on a 1:1 staff to patient ratio and averaged 6 weeks. During the period of analysis, only Aboriginal people were trained and there were no withdrawals from the training program. However high infection rates and technique failure once at home, resulted in higher staff to patient ratios and more frequent remote community visits. Consumables costs were comparatively high and possibly reflect the greater uptake of automated peritoneal dialysis (APD) which was one third more expensive than continuous ambulatory peritoneal dialysis (CAPD). Capital costs were only allocated for the training component.

The average annual per patient cost in the first year were $87,250 with costs in the second and subsequent years at $58,489 (Fig. 4).

## Discussion

The mismatch between service demand and service location in the NT, has largely been due to the perceived expense *to the health service* of providing dialysis in remote rather than urban locations. The challenges associated with remote service delivery, such as inadequate infrastructure, skilled workforce shortages and limited transport options, do incur higher costs. This is consistent with international evidence [12]. In Australia, states with similar Indigenous populations living remotely (Far north Queensland and northern Western Australia) acknowledge that these challenges limit patients’ access to some dialysis models of care [39–41].

DxMoC1 hub services had higher costs, largely due to their role in providing support and oversight of several program areas and facilities. Co-location of services provided some cost efficiencies to the DoH, however the equitable allocation of expenditure according to service usage was not always clear. Most urban services also had lower annual patient attendance rates, (75–87%) compared to rural and remote services (over 90%). Our experience suggests that patients who are forced to relocate to access urban services have poorer attendance.

The PP (contracted) DxMoC1 service in Central Australia was the least expensive of all the urban and rural staffed facilities. The PP service routinely achieved nearly 100% attendance. In part this was due to the contract conditions specifying reimbursement according to attendance. This in turn, motivated management and staff to support patients to attend. Additionally, the contracted service had limited responsibilities for broader renal program delivery compared to the hub service.

An expectation that services in rural and remote locations cost considerably more proved only partially true. The cost drivers for these locations related to staffing models and subsequent salary and travel costs. Services that employed local staff (DxMoC2) had similar overall costs to urban services, while services that employed a fly-in fly-out model (DxMoC3), spent considerably more on salaries, allowances and staff transport.

The DxMoC4 community-controlled service was generally perceived as a high cost model of care, primarily because services were delivered from multiple, small, low volume sites and because the model offered a comprehensive program of social and cultural support, unlike the other models. Despite this and the higher per treatment costs, our analysis found the service was *less* expensive than the cost of delivering care at a single remote site (DxMoC3). During the period of analysis, DxMoC3 and the multiple locations of DxMoC4 were never at capacity and patient numbers fluctuated over the year. However, adherence to the prescribed number of treatments for individuals attending remote services (and rural services) was better than the urban services, averaging over 90%. We surmised that these services, perhaps by virtue of their smaller size and proximity to community and family, facilitated closer relationships and provided opportunities for more personalised care, resulting in better attendance.

The first year costs for both self-care therapies (HD and PD) were higher than in other cost studies (national/international), primarily due to more lengthy training programs and associated staff costs [17, 42, 43]. The selfcare training component made up 68% of first year costs for home HD compared to 38% for PD. The programs were particularly sensitive to treatment and patient numbers. However, despite the additional requirements of delivering a self-care program in remote areas, including facility management, the costs of the home HD program were less than care in staffed facilities for both the first and subsequent years. The ongoing costs for home HD were similar to other studies in Australia and overseas [17, 42].

Our study found the PD program was considerably more expensive than the home HD program, driven by the much higher PD consumable costs, despite the additional machinery and maintenance costs of HD. PD costs mirrored early cost studies in Australia [44] but not more recent studies undertaken by various states in Australia [17].

### Strengths and limitations

To our knowledge this is the first comprehensive exploration of dialysis services costs in remote locations in Australia. The study took a micro-costing, top down approach and applied a rigorous methodology in the analysis of the data. We used the best available data from multiple sources and went to significant lengths to locate missing information.

However, we acknowledge that our analysis also has some limitations. We have focussed on recurrent costs of each ‘outpatient’ service for maintenance dialysis and did not include in-patient or in-centre dialysis related costs. There were challenges with incomplete data and separation of costs where service costs were centralised, single cost centres for different types of activities existed and supply lines or facilities were shared. Assumptions and calculations were based on advice from corporate, financial and clinical staff working in these areas.

## Conclusions

Given the demand for services closer to home, where “home” is in a remote location, our study identifies opportunities for cost minimisation of dialysis services. Models of care embedded within communities, employing local staff, achieve cost efficiencies by reducing personnel and travel costs. With high levels of unemployment in remote communities in the NT (Aboriginal labour force participation rate 37.3% [45]) there are opportunities to train local people in a variety of roles to support community dialysis. Community based services are also likely to be more acceptable to patients with emerging evidence in the NT suggesting a strong link between dialysis attendance rates and the proximity of a facility to a patient’s home community. We know attendance rates significantly influence the annual per patient cost of a service but also have substantial impacts on other health service utilisation such as unplanned hospital admissions or medical evacuations and can increase morbidity. These costs can be quantified but have not been fully explored nor encompassed in cost studies to date [24, 46]. The true costs of the provision of dialysis services are likely to be underestimated.

Our cost study provides the basis for the next component of this research which evaluates the broader health service utilisation costs (hospitalisations, medical evacuations, emergency department presentations), associated with a patient’s preference for a model of care. The relationship between health service uptake, facility size and location will be explored as will the costs of transitioning between models of care, particularly for the self-care therapies. The addition of these annual hospital costs to the model of care they attend, will provide a more accurate reflection of service delivery costs per patient per location and model of care.

## Additional files

Additional file 1: Geographic classification of study sites compared to local terminology. Comparison of study location designations and the official Australian classifications for remoteness. (DOCX 13 kb)

Additional file 2: “What is the Purple House?”. Description of data: Additional information on development and role of Purple House. (DOCX 2872 kb)

## Data Availability

Access to the data analysed in this study is governed by the data custodians and is currently not publicly available due to confidentiality concerns. Data may be made available upon request and subject to data owner approvals.

## Abbreviations

ANZDATA: Australian & New Zealand Dialysis and Transplant Registry
APD: Automated peritoneal dialysis
CA: Central Australia
CAPD: Continuous ambulatory peritoneal dialysis
CPT: Cost per treatment
DoH: Department of Health
DPMQ: Dispensed Price for Maximum Quantity
DxMoC: Dialysis models of care
FIFO: Fly-in Fly-out
HD: Haemodialysis
HPI: Health Price Index
MBS: Medicare Benefits Schedule
NEP: National Efficient Price
NT: Northern Territory
PCA: Patient Care Assistant
PD: Peritoneal Dialysis
PH: Purple House
PPT: Price per treatment
pt.: Patient
TE: Top End
tr: Treatment
