# Supplementary material for: Cost of dialysis therapies in rural and remote Australia – a micro-costing analysis
Source: BMC Nephrol. 2019 Jun 25;20:231. doi: 10.1186/s12882-019-1421-z (PMC6593509; doi:10.1186/s12882-019-1421-z)
Supplement: Supplementary file 1 — Geographic classification of study sites compared to local terminology. Comparison of study location designations and the official Australian classifications for remoteness. (DOCX 13 kb) [file 12882_2019_1421_MOESM1_ESM.docx]

Additional File 1 Geographic classification of study sites compared to local terminology

| Locality | DxMoC Study designations | Service Population | Km to Hub unit | *ABS designation |
| --- | --- | --- | --- | --- |
| Site 1 - TE | DxMoC1 - urban | 170,000 | Main hub | outer regional |
| Site 2 - TE | DxMoC1- urban | 170,000 | 30 | outer regional |
| Site 3 - CA | DxMoC1- urban | 25,000 | Hub | remote |
| Site 4 - CA | DxMoC1- urban | 25,000 | 0 | remote |
|  |  |  |  |  |
| Site 5 - TE | DxMoC2 - rural | 6,300 | 320 | remote |
| Site 6 - CA | DxMoC2 - rural | 2,991 | 508 | remote |
|  |  |  |  |  |
| Site 7 - TE | DxMoC3 - remote | 2453 | 80 (by air) | very remote |
|  |  |  |  |  |
| Site 8 - CA | DxMoC4 - remote | 454 | 521 | very remote |
| Site 9 - CA | DxMoC4 - remote | 687 | 293 | very remote |
| Site 10 - CA | DxMoC4 - remote | 625 | 131 | very remote |
| Site 11 - CA | DxMoC4 - remote | 669 | 890 | very remote |
| Site 12 - WA | DxMoC4 - remote | 474 | 1000 | very remote |
| Site 13 - WA | DxMoC4 - remote | 216 | 850 | very remote |
| Site 14 - CA | DxMoC4 - remote | 643 | 85 | very remote |
