# Supplementary material for: Cost of dialysis therapies in rural and remote Australia – a micro-costing analysis
Source: BMC Nephrol. 2019 Jun 25;20:231. doi: 10.1186/s12882-019-1421-z (PMC6593509; doi:10.1186/s12882-019-1421-z)
Supplement: Supplementary file 2 — “What is the Purple House?”. Description of data: Additional information on development and role of Purple House. (DOCX 2872 kb) [file 12882_2019_1421_MOESM2_ESM.docx]

**Additional File 2: What is the “*Purple House?”***

What is now the ‘Purple House’ was known until recently as *Western Desert Nganampa Walytja Palyantjaku Tjutaku Aboriginal Corporation.* A registered charity with Deductible Gift Recipient Status (DGR), the organization was formally incorporated in the NT in 2003. Initially it provided social and cultural support plus a limited respite dialysis service for remote Aboriginal patients (and families) of the Western Desert homelands area centred on the very remote communities of Kintore and Kiwirrkurra, 500+ kilometres east of Alice Springs. In the preceding years it had become clear to those Aboriginal patients, their families and supporters that Governments, Health Departments and politicians would neither extend staffed services to their remote homelands, nor concede that health itself was dependent on patients maintaining connections to family, community and country.

*So together with their art centre, Papunya Tula Artists, Sotheby’s auction house and Curator Hetti Perkins from the Art Gallery of NSW, they painted some incredibly beautiful paintings. The paintings were auctioned at the Art Gallery of NSW in November 2000 and raised over a million dollars in a single night.*

*It was a million dollars of independent money, not government money. This was their chance. A chance to create their own model of care . A model that was safe, sustainable, with cultural priorities at the front and centre. It needed to be community-controlled with real ownership. [Brown 2018:105]*

Thus the *Western Desert Nganampa Walytja Palyantjaku Tjutaku Aboriginal Corporation,* lately the formally re-named ‘Purple House’ arose. Things have not looked back since then!

The Purple House is governed by a Board of 12 Aboriginal Directors comprising Traditional Landowners and community Elders. The organization has a Vision to:

*Improve the lives of people with renal failure, reunite families and reduce the impact of kidney disease in our communities.*

The organisation seeks funding from government, non-government and other philanthropic organisations in the form of service level agreements, donations and in-kind support to assist Aboriginal people who have been relocated for treatment. It attracts skilled professionals as well as volunteers who contribute to its many activities and has now built up a multidisciplinary team of paid staff and volunteers. All dialysis is provided by qualified and suitably trained nursing staff and the organisation is accredited with all required Australian Health Care bodies and meets the required legislation for health service delivery. The Purple House has established programs providing social support for those in the urban area, coordinating respite visits to a patient’s remote home community, with or without dialysis; and the provision of on-community dialysis services.

In the nearly 20 years since it was established the Purple House has expanded exponentially with programs, activities and services stretching the length and breadth of the Northern Territory and beyond as communities seek out alternative ways to deal with the kidney disease that is still rampant in these remote communities. Services are also now being provided to patients and families in the remote regions of neighbouring states: South Australia and Western Australia.


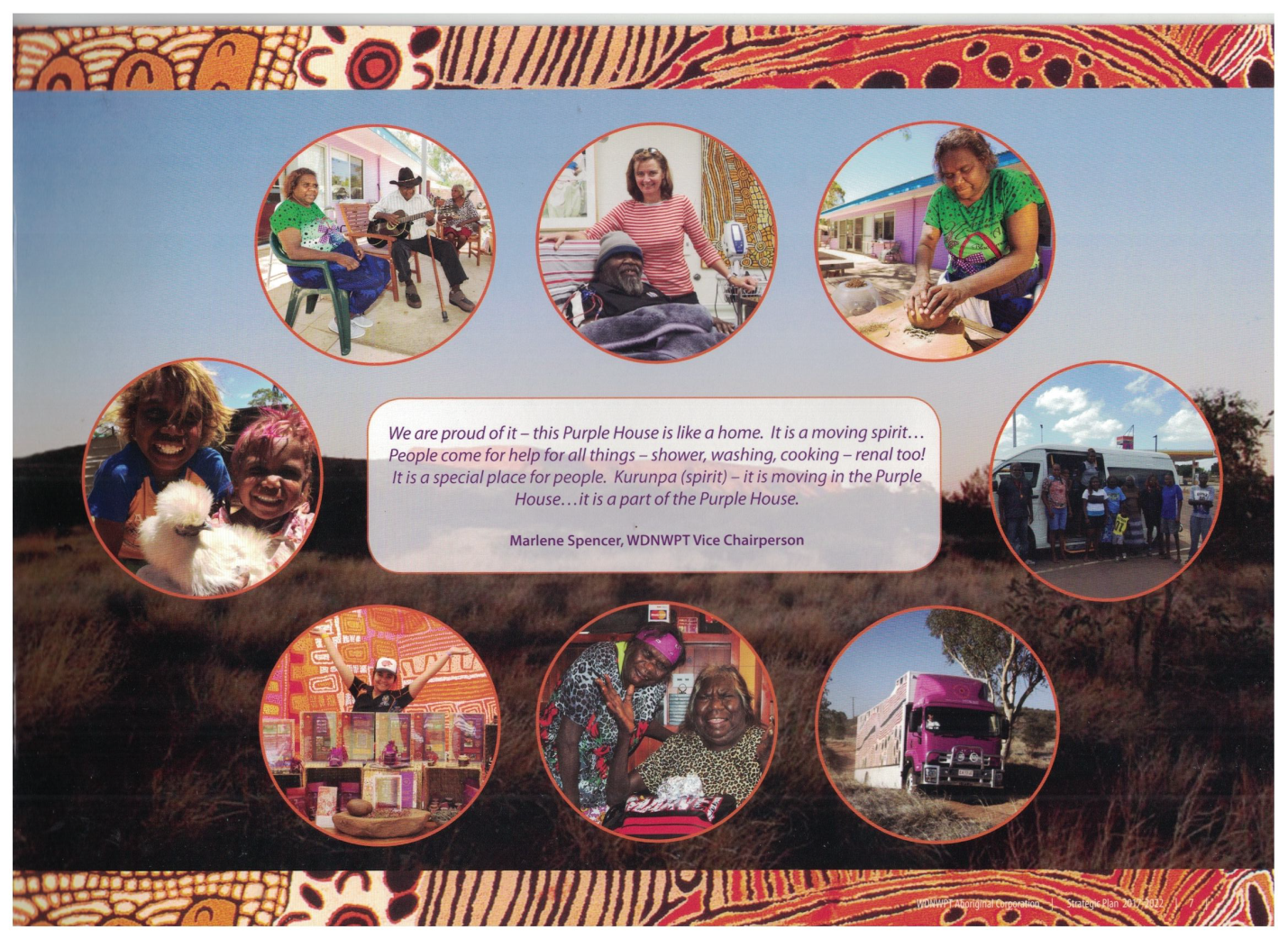


Source: WDNWPT: **Strategic Plan 2017-2022**. Alice Springs, NT: Western Desert Nganampa Walytja Palyantjaku Tjutaku Aboriginal Corporation; 2017. Page 7
